# Diagnosis of glomus tumor and preoperative mapping with ultrasonography^⋆^

**DOI:** 10.1016/j.abd.2023.02.013

**Published:** 2024-08-31

**Authors:** Athos Paulo Santos Martini, Ariel Córdova Rosa, Marcelo Rigatti, Gabriella Di Giunta Funchal, Matheus Hariel Colin Torres, Matheus Alves Pacheco

**Affiliations:** Hospital Universitário da Universidade Federal de Santa Catarina, Florianópolis, SC, Brazil

*Dear Editor,*

The diagnosis of glomus tumors remains a challenge and there is debate about the most accurate imaging test for diagnosis.

A 35-year-old male patient was referred to the dermatology outpatient clinic for an ultrasound evaluation of the fourth finger. He had had moderate periungual pain in the affected finger for the last six months, which worsened with local pressure.

In the clinical evaluation, no changes were identified in the nail transillumination examination and there was no worsening with exposure to cold. Love's pin test was positive.

The patient had undergone magnetic resonance imaging (MRI) weeks before, but no tumor was identified at the site of the complaint. Due to clinical suspicion, he was referred for evaluation with high-frequency ultrasound.

The examination was performed with a 22-MHz linear transducer, disclosing a hypoechoic nodular lesion, with regular, well-defined contours and intensely vascularized on Color Doppler, measuring 2.0 × 1.5 mm in the nail matrix, and discrete bone remodeling (Figure 1, Figure 2). Ultrasonographic mapping of the lesion and surgical resection were performed (Fig. 3). Histopathology confirmed the identification of the tumor (Fig. 4).

**Figure 1:**
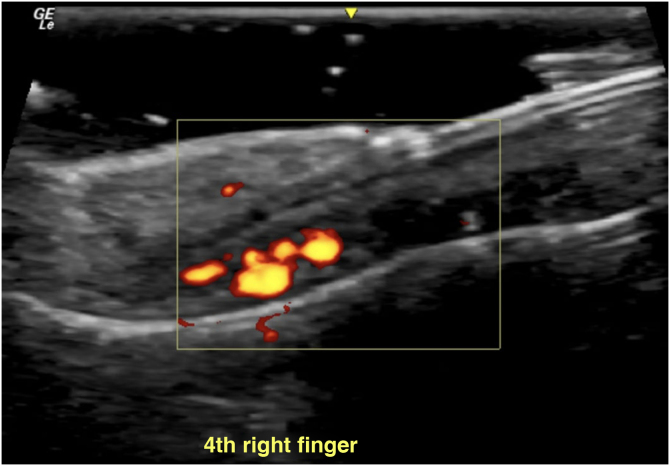
Increased vascularization in the nail matrix ‒ longitudinal axis.

**Figure 2:**
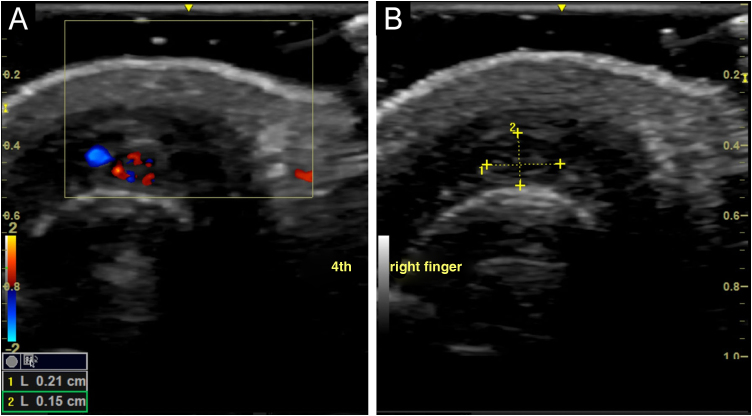
(A) Hypoechoic vascular lesion in the nail matrix – transversal axis. (B) Lesion measuring 2.1 × 1.5 mm ‒ transversal axis.

**Figure 3:**
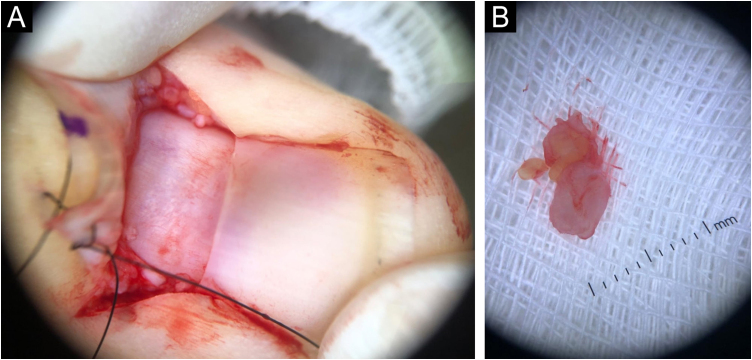
Nail surgery. (A) Exposure of the nail matrix. (B) Resected tumor.

**Figure 4:**
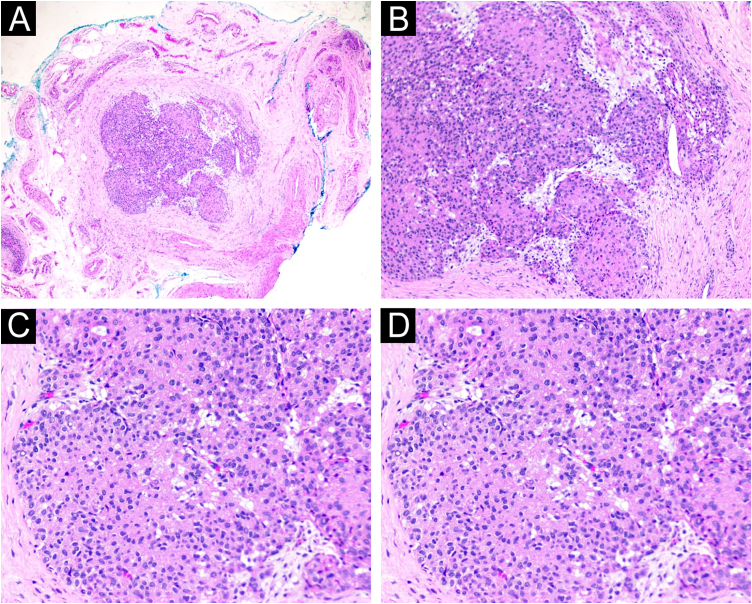
(A) Tumor nodule well demarcated by a fibrous pseudocapsule (Hematoxylin & eosin, ×40). (B) Proliferation of round cells with eosinophilic cytoplasm surrounded by amphophilic stroma surrounding branched vessel (Hematoxylin & eosin, ×100). (C) Proliferation of round cells with eosinophilic cytoplasm (Hematoxylin & eosin, ×200). (D) Amphophilic stroma surrounding branched vessels (Hematoxylin & eosin, ×200).

Originating from the glomus bodies, which control blood pressure and temperature by regulating peripheral cutaneous circulation, the glomus tumor is described as a rare benign tumor with a low potential for malignancy.^1^

The clinical manifestation of this type of tumor is represented by the hallmark triad: Love's pin test (pressure applied to the suspicious area with a pin head causes intense pain), Hildreth's Test (pain and reduced sensitivity after applying a tourniquet proximal to the lesion) and the sensitivity test to cold (pain amplification on exposure to cold). Transillumination testing can help determine tumor size.^2^

Because it is not usually possible to detect nail tumors through inspection and palpation, they are sometimes misdiagnosed. Investigation using complementary imaging tests can help define the diagnosis.^3^

Currently, there is controversy regarding the diagnostic value of MRI for glomus tumors. Previous studies have demonstrated good diagnostic value, while others suggest poor value.^4^

A useful resource for diagnosing glomus tumors is ultrasound, especially high-frequency ultrasound, which can precisely determine tumor location. The ultrasound is non-invasive and can easily characterize tumors as small as 3 mm in diameter in any cross-section in the hands of trained operators.^3^, ^5^, ^6^

Magnetic resonance imaging depends on intravenous contrast and is not sensitive enough for tumors smaller than 3 mm. Meanwhile, the ultrasound, in addition to not depending on these factors, offers preoperative information about the tumor to guide the procedure and improve results, demonstrating the potential usefulness of this exam in the evaluation of glomus tumors.^7^

On ultrasound, glomus tumors are described as well-circumscribed hypoechoic masses. Almost all previously reported cases have shown signs of rich internal blood flow within the hypoechoic area.^8^

Preoperative color Doppler ultrasound can precisely define the following characteristics: tumor location and size, distance to the surface, margins, presence or absence of capsule, and the relationship between the internal circulation and surrounding tissues – all important parameters for surgical evaluation.^9^

Only surgical resection has been shown to be effective in treating glomus tumors.^10^

It was believed that MRI was the best test to help find small nail tumors, but with the evolution of ultrasound, especially high-frequency ultrasound, this idea came to be questioned. Ultrasound has proven to be an important diagnostic tool for nail tumors, as good as or even better than MRI.

In the present case, the authors were able to locate a small tumor that was not identifiable on MRI and precisely determined the surgical parameters using ultrasound. The aim of the present study was to increase diagnostic possibilities, but more studies are needed to determine the reference imaging tests for nail tumors.

## Financial support

None declared.

## Authors' contributions

Athos Paulo Santos Martini: Design and planning of the study; drafting and editing of the manuscript or critical review of important intellectual content.

Ariel Córdova Rosa: Design and planning of the study.

Marcelo Rigatti: Design and planning of the study.

Gabriella Di Giunta Funchal: Design and planning of the study.

Matheus Hariel Colin Torres: Drafting and editing of the manuscript or critical review of important intellectual content.

Matheus Alves Pacheco: Drafting and editing of the manuscript or critical review of important intellectual content.

## Conflicts of interest

None declared.
